# Incidence and Outcomes Associated With *Clostridium difficile* Infections: A Systematic Review and Meta-analysis

**DOI:** 10.1001/jamanetworkopen.2019.17597

**Published:** 2020-01-08

**Authors:** Alexandre R. Marra, Eli N. Perencevich, Richard E. Nelson, Matthew Samore, Karim Khader, Hsiu-Yin Chiang, Margaret L. Chorazy, Loreen A. Herwaldt, Daniel J. Diekema, Michelle F. Kuxhausen, Amy Blevins, Melissa A. Ward, Jennifer S. McDanel, Rajeshwari Nair, Erin Balkenende, Marin L. Schweizer

**Affiliations:** 1 Carver College of Medicine, Department of Internal Medicine, University of Iowa, Iowa City; 2 Division of Medical Practice, Hospital Israelita Albert Einstein, São Paulo, Brazil; 3 Center for Access and Delivery Research and Evaluation, Iowa City VA Health Care System, Iowa City, Iowa; 4 Veterans Affairs Salt Lake City Health Care System, Salt Lake City, Utah; 5 Department of Internal Medicine, University of Utah, Salt Lake City; 6 Big Data Center, China Medical University Hospital, Taichung City, Taiwan; 7 Division of Epidemiology and Community Health, University of Minnesota, Minneapolis; 8 Ruth Lilly Medical Library, Indiana University School of Medicine, Indianapolis

## Abstract

**Importance:**

An understanding of the incidence and outcomes of *Clostridium difficile* infection (CDI) in the United States can inform investments in prevention and treatment interventions.

**Objective:**

To quantify the incidence of CDI and its associated hospital length of stay (LOS) in the United States using a systematic literature review and meta-analysis.

**Data Sources:**

MEDLINE via Ovid, Cochrane Library Databases via Wiley, Cumulative Index of Nursing and Allied Health Complete via EBSCO Information Services, Scopus, and Web of Science were searched for studies published in the United States between 2000 and 2019 that evaluated CDI and its associated LOS.

**Study Selection:**

Incidence data were collected only from multicenter studies that had at least 5 sites. The LOS studies were included only if they assessed postinfection LOS or used methods accounting for time to infection using a multistate model or compared propensity score–matched patients with CDI with control patients without CDI. Long-term-care facility studies were excluded. Of the 119 full-text articles, 86 studies (72.3%) met the selection criteria.

**Data Extraction and Synthesis:**

Two independent reviewers performed the data abstraction and quality assessment. Incidence data were pooled only when the denominators used the same units (eg, patient-days). These data were pooled by summing the number of hospital-onset CDI incident cases and the denominators across studies. Random-effects models were used to obtain pooled mean differences. Heterogeneity was assessed using the *I*^2^ value. Data analysis was performed in February 2019.

**Main Outcomes and Measures:**

Incidence of CDI and CDI-associated hospital LOS in the United States.

**Results:**

When the 13 studies that evaluated incidence data in patient-days due to hospital-onset CDI were pooled, the CDI incidence rate was 8.3 cases per 10 000 patient-days. Among propensity score–matched studies (16 of 20 studies), the CDI-associated mean difference in LOS (in days) between patients with and without CDI varied from 3.0 days (95% CI, 1.44-4.63 days) to 21.6 days (95% CI, 19.29-23.90 days).

**Conclusions and Relevance:**

Pooled estimates from currently available literature suggest that CDI is associated with a large burden on the health care system. However, these estimates should be interpreted with caution because higher-quality studies should be completed to guide future evaluations of CDI prevention and treatment interventions.

## Key Points

**Question:**

What is the incidence of hospital-onset *Clostridium difficile* infection (CDI) and its associated length of stay?

**Findings:**

This systematic review and meta-analysis of 13 studies using patient-days as the denominator found that the incidence of hospital-onset CDI was 8.3 cases per 10 000 patient-days. Among propensity score–matched studies of the length of stay, the mean difference in length of stay between patients with and those without CDI varied from 3.0 to 21.6 days.

**Meaning:**

Pooled estimates from currently available literature suggest that CDI is associated with a large burden on the US health care system.

## Teaser

This systematic review and meta-analysis of 13 studies analyzes the incidence of *Clostridium difficile *infection and its associated hospital length of stay in the United States.

## Introduction

*Clostridium difficile* (also known as *Clostridioides difficile*) is the most common pathogen causing health care–associated infections in the United States, accounting for 15% of all such infections.^1^ A Centers for Disease Control and Prevention report on antibiotic resistance threats categorized *C difficile* as an urgent threat.^2^ Antibiotic treatment for *C difficile* infection (CDI) is often followed by recurrent infection, leading to nontraditional treatments, such as fecal transplant and oral administration of nontoxigenic *C difficile* spores.^3,4^

Information about the burden of CDI in the United States could inform investments in prevention and treatment interventions. This information should include the incidence of CDI, how this incidence has changed over time, and poor outcomes associated with CDI. Although prior studies have shown that CDI is associated with poor outcomes, such as recurrence, long hospital length of stay (LOS), mortality, and high treatment costs, these results vary by study location and patient population.^2,5^ In addition, many current estimates of the poor outcomes and costs associated with CDI do not take into account the underlying severity of illness among patients who develop CDI and may overestimate the true attributable outcomes.^6^

To address gaps in our understanding of the current burden associated with CDI in the United States, we conducted a systematic literature review of studies conducted in the United States and published after 2000 that evaluated the incidence of CDI and associated LOS. The goals were to describe the recent incidence of CDI and to evaluate LOS attributable to CDI.

## Methods

### Search Strategy

This systematic review and meta-analysis was conducted according to the Preferred Reporting Items for Systematic Reviews and Meta-analyses (PRISMA)^7^ and Meta-analysis of Observational Studies in Epidemiology (MOOSE)^8^ reporting guidelines. An experienced health sciences librarian (A.B.) conducted systematic searches in MEDLINE via Ovid, Cochrane Library Databases via Wiley, Cumulative Index of Nursing and Allied Health Complete via EBSCO Information Services, Scopus, and Web of Science to identify articles published from the inception of the database to February 2019. Citations published before 2000 were excluded. A combination of keywords and subject headings were used for “*Clostridium difficile*,” “length of stay,” and “incidence.” The full search strategies can be found in eAppendix 1 in the Supplement.

### Inclusion and Exclusion Criteria

Publications were included if they evaluated the incidence of CDI or LOS associated with CDI. Studies were excluded if they did not contain original data, did not have a control group, were published outside the United States, were published in a language other than English, or were published before 2000. The year 2000 was chosen as the beginning of this systematic literature review because that was when the epidemic BI/NAP1/027 strain of *C difficile* emerged, after which CDI increased in prevalence and became less responsive to treatment.^4^ We excluded studies if they assessed only a specific subset of patients, unless that population could be categorized as 1 of the following subsets: immunocompromised patients, patients in the intensive care unit, patients with cancer, patients with end-stage renal disease, patients undergoing hemodialysis, surgical patients, solid-organ transplant recipients, patients with high-risk gastrointestinal conditions, or peripartum women. We excluded studies with a study period of less than 1 year. We also excluded studies of long-term care facilities. Incidence data were collected only from multicenter studies that had at least 5 sites, because single-site or small studies may be biased by outbreaks or other local conditions. We included incidence studies with denominators of patient-days or person-years, known timing of the CDI such as after surgery or after admission (ie, hospital onset [HO]), or exclusion of patients with a history of CDI.

Studies were included in the LOS analysis only if they provided data on postinfection LOS, if they used methods accounting for time to infection using a multistate model, or if propensity score–matched patients with CDI were compared with uninfected controls.^5,9^ Studies were excluded if they did not have an uninfected control group or a denominator that included patients without CDI.

### Data Extraction and Quality Assessment

Titles and abstracts of all articles were screened to assess inclusion criteria. Two of 9 independent reviewers (M.L.S., M.A.W., M.F.K., H.-Y.C., M.L.C., L.A.H., D.J.D., A.R.M., and E.N.P.) abstracted data for each article. Reviewers resolved disagreements by consensus.

The reviewers abstracted data on study design, study population, setting and years, inclusion and exclusion criteria, number of patients included, description of control group, definition of CDI, outcomes (eg, incidence and LOS), and an assessment of the potential risk of bias. Risk of bias was assessed using the Downs and Black scale.^10^ Reviewers followed all questions from this scale as written except for question 27 (a single item on the Power subscale, which was scored 0-5), which was changed to a yes or no. Two of us (A.R.M. and M.L.S.) performed component quality analysis independently, reviewed all inconsistent assessments, and resolved disagreements by consensus.^11^

### Statistical Analysis

 Data analysis was performed in February 2019. Excel spreadsheet software version 2007 (Microsoft Corp) and RevMan statistical software version 5.3 (Cochrane Community) were used for statistical analysis. Incidence data were pooled only when the denominators used the same units (eg, patient-days). These data were pooled by summing the number of HO-CDI incident cases and the denominators across studies. Pooled incidence was reported as the number of incident cases per the given denominator (eg, 10 000 patient-days).^12^ No *P* values were calculated.

## Results

Of the 34 775 articles identified (Figure), 119 were full-text articles, and 86 (72.3%) of those articles met the selection criteria and were included in the systematic literature review.^13,14,15,16,17,18,19,20,21,22,23,24,25,26,27,28,29,30,31,32,33,34,35,36,37,38,39,40,41,42,43,44,45,46,47,48,49,50,51,52,53,54,55,56,57,58,59,60,61,62,63,64,65,66,67,68,69,70,71,72,73,74,75,76,77,78,79,80,81,82,83,84,85,86,87,88,89,90,91,92,93^ Among these, 66 articles evaluated incidence,^13,14,15,16,17,18,19,20,21,22,23,24,25,26,27,28,29,30,31,32,33,34,35,36,37,38,39,40,41,42,43,44,45,46,47,48,49,50,51,52,53,54,55,56,57,58,59,60,61,62,63,64,65,66,67,68,69,70,71,72,73,74,75,76,77,78^ and 20 articles evaluated LOS.^16,54,66,79,80,81,82,83,84,85,86,87,88,89,90,91,92,93,94,95^ One-fifth of the studies that assessed LOS (4 studies)^84,87,91,94^ scored 18 or more points of the 28 points possible on the Downs and Black scale^10^ and, thus, were considered to be of higher quality.

**Figure.**
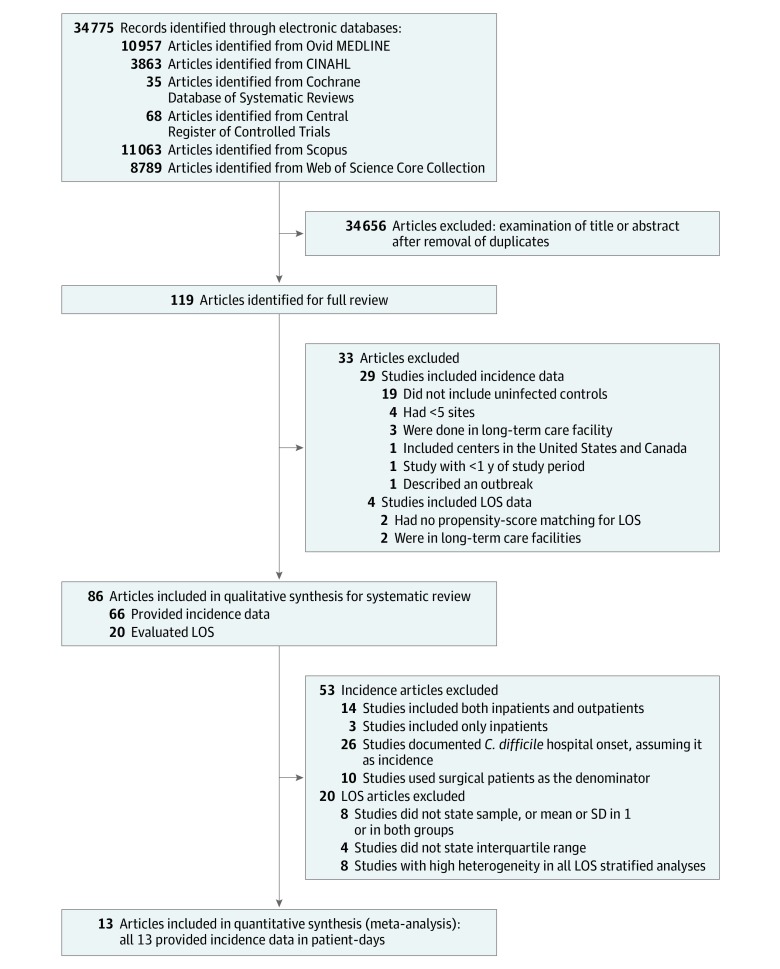
Literature Search for Articles That Evaluated Incidence and Length of Stay (LOS) Associated With *Clostridium difficile* Infection CINAHL indicates Cumulative Index of Nursing and Allied Health.

### Incidence of CDI Calculated Using Patient-Days (13 Studies)

Sixty-six studies^13,14,15,16,17,18,19,20,21,22,23,24,25,26,27,28,29,30,31,32,33,34,35,36,37,38,39,40,41,42,43,44,45,46,47,48,49,50,51,52,53,54,55,56,57,58,59,60,61,62,63,64,65,66,67,68,69,70,71,72,73,74,75,76,77,78^ measured CDI incidence. Thirteen of those 66 studies^13,14,15,16,17,18,19,20,21,22,23,24,25^ used patient-days as the denominator (Table 1). Among these studies, the CDI incidence varied from 2.8 CDI cases per 10 000 patient-days^22^ to 15.8 CDI cases per 10 000 patient-days.^20^ Three studies^13,17,23^ were conducted by the Centers for Disease Control and Prevention. Three studies^17,18,21^ were done in New York State. One study^24^ from Southern California found that the incidence of community-onset, health care facility (HCF)–associated CDI (11.1 cases per 10 000 patient-days) was almost 2-fold higher than that for HO, HCF-associated CDI (6.8 cases per 10 000 patient-days). The pooled incidence of HO-CDI among the 13 studies^13,14,15,16,17,18,19,20,21,22,23,24,25^ (Table 1) that used patient-days as the denominator was 8.3 CDI cases per 10 000 patient-days. Four studies^13,15,18,21^ included more than 100 facilities.

**Table 1.** Multicenter Studies (≥5 Sites) That Evaluated *Clostridium difficile* Infection Incidence Calculated Using Patient-Days

Source	Data Set	Study Period	Facilities or Hospitals, No.	*C difficile* Definition	Incidence
Archibald et al,^13^ 2004	CDC’s National Nosocomial Infections Surveillance	1987-2001	90-340 Hospitals depending on year	CDC definition	Teaching hospital intensive care units, 5.1 cases/10 000 patient-days; nonteaching hospital intensive care units, 4.4 cases/10 000 patient-days
Burger et al,^14^ 2006	Veterans’ Health Administration East Coast Infection Control Council hospitals	Q3 1999 to Q4 2002	32	CDC definition	7.9 Cases/10 000 patient-days
Campbell et al,^15^ 2009	State of Ohio	January 1, 2006, to December 31, 2006	210	*ICD-9* code, laboratory tests, clinical findings	6.4-7.9 Cases/10 000 patient-days
Dubberke et al,^16^ 2010	Hospitals in CDC Epicenter Network	July 2000 to June 2006	5	*C difficile* toxin assay results and the *ICD-9* code	HO, HCF–associated cases: 7.0 cases/10 000 patient-days in 2001 and 8.5 cases/10 000 patient-days in 2006
Gase et al,^17^ 2013	New York State National Healthcare Safety Network	July-December 2009	30	Clinical findings, laboratory tests	9.66 Cases/10 000 patient-days (95% CI, 9.21-10.1 cases/10 000 patient-days)
Haley et al,^18^ 2014	New York hospital discharge billing records	2010	124	Clinical findings, laboratory tests	11.6 Cases/10 000 patient-days
Kim et al,^19^ 2008	Pediatric Health Information System Database	2001-2006	22	*ICD-9 *code, billing charge for *C difficile* toxin assay, and an initial dose of *C difficile* antibiotic therapy in the period of 1 d before to 2 d after *C difficile* toxin assay	2001, 4.4 Cases/10 000 patient-days; 2006, 6.5 cases/10 000 patient-days
Kamboj et al,^20^ 2012	Comprehensive Cancer Center’s Infection Control Group Network of Patients with Cancer or Hematopoietic Stem Cell Transplant	2010-2011	11	Laboratory tests and *C difficile* surveillance	HO *C difficile* infection, 15.8 cases/10 000 patient-days
McDonald et al,^21^ 2012	3 State-led programs (Illinois, Massachusetts, New York)	2008-2011	711	Clinical findings, laboratory tests	7.4 Cases/10 000 patient-days
Miller et al,^22^ 2011	Duke Infection Control Outreach Network	2005-2009	28	Infection preventionist evaluated surveillance or diagnosis	2.8 Cases/10 000 patient-days
Sohn et al,^23^ 2005	Hospitals in CDC Epicenter Network	2000-2003	7	Clinical findings, laboratory tests, and CDC surveillance of *C difficile*	12.1 Cases/10 000 patient-days (mean range, 3.1-25.1 cases/patient-days); 7.4 cases/1000 admissions (mean range, 3.1-13.1 cases/1000 admissions)
Tartof et al,^24^ 2014	Kaiser Permanente Southern California health care system	2011-2012	14	Laboratory tests: polymerase chain reaction	Community-onset, HCF-associated, 11.1 cases/10 000 patient-days; HO, HCF-associated, 6.8 cases/10 000 patient-days
Zilberberg et al,^25^ 2011	CareFusion clinical research database	January 2007 to June 2008	85	Laboratory tests	6.3 Cases/10 000 patient-days

Abbreviations: CDC, Centers for Disease Control and Prevention; HCF, health care facility; HO, hospital onset; *ICD-9*, *International Classification of Diseases, Ninth Revision*; Q, quarter.

The definitions of *C difficile* used to identify cases varied. Three studies^17,18,21^ used clinical findings and results of laboratory tests for *C difficile*, 3 studies^13,14,23^ used the Centers for Disease Control and Prevention surveillance definition to identify *C difficile*, 2 studies^20,22^ applied infection preventionist evaluations for *C difficile* surveillance, and 2 studies^24,25^ used only results of laboratory tests for *C difficile*. The remaining studies used a variety of ways to identify CDI, including *International Classification of Diseases, Ninth Revision *(*ICD-9*) codes or other billing codes,^15,16,19^ laboratory test results,^15,16,20,23^ clinical findings,^15,23^ and initial doses of *C difficile* antibiotic therapy.^19^ When we examined incidence by time period, we found that the early studies from 2000 to 2008 had a range from 2.8 to 12.2 CDI cases per 10 000 patient-days, studies from 2008 to 2009 had a range from 6.3 to 9.6 CDI cases per 10 000 patient-days, and the later studies after 2010 reported a range from 6.8 to 15.8 CDI cases per 10 000 patient-days (Table 1).

### Incidence of CDI Calculated Using Person-Years (17 Studies)

Fourteen studies^26,27,28,29,30,31,32,33,34,35,36,37,38,39^ included both inpatients and outpatients (Table 2), reflected in a denominator of person-years in 8 studies.^27,28,29,30,32,34,36,39^ Seven of those 14 studies^27,28,29,30,32,34,39^ used only *ICD-9 *codes to define CDI. In a study^36^ of adult and adolescent patients with HIV/AIDS that included more than 100 hospitals, during 10 years of study, the peak incidence of CDI was 9.59 cases per 1000 person-years among patients with clinical AIDS. A study^28^ of the Armed Forces Health Surveillance Center in Maryland over the course of 12 years found the incidence of community-associated CDI to be 5.5 cases per 100 000 person-years. In a study^29^ evaluating the annual incidence of CDI and multiply recurrent CDI per 1000 person-years, the incidences increased by 42.7% and 188.8%, respectively, during a decade (2001-2012) in the United States. In another study^30^ with 12 years of data from 5 administrative databases, elderly people (ie, aged >65 years) had a CDI rate of 677 cases per 100 000 person-years. In contrast, a managed-care organization in Colorado found that the CDI incidence in 2007 was 14.9 CDI cases per 10 000 patient-years.^32^ These studies were too diverse to pool together into 1 estimate.

**Table 2.** Multicenter Studies (≥5 Sites) That Evaluated *Clostridium difficile* Infection Incidence Calculated Using Person-Years

Source	Data Set	Study Period	Facilities or Hospitals, No.	*C difficile* Definition	Incidence
Denominator: geographic population (inpatient and outpatient)					
Chernak et al,^26^ 2005	Philadelphia, Pennsylvania, and surrounding 4 counties	2004-2005	Not stated	Clinical diagnosis	Community-associated, 7.6 cases/100 000 population
Dubberke et al,^27^ 2016	Medicare Chronic Condition Warehouse (5% random sample)	2009	5% Random sample	*ICD-9*	Overall incidence of CDI, 677 cases/100 000 persons
Gutiérrez et al,^28^ 2013	Defense Medical Surveillance Center, Armed Forces Health Surveillance Center, US Department of Defense, Silver Spring, Maryland	1998-2010	Not stated	*ICD-9*	*C difficile–*associated disease incidence, 13.2 cases/100 000 person-years; community-associated, 5.5 cases/100 000 person-years; health care *C difficile*–associated disease, 1.3 cases/1000 hospitalizations
Ma et al,^29^ 2017	OptumInsight Clinformatics Database	2001-2012	38 911 718 Commercially insured patients	*ICD-9*	Annual incidence of CDI and multiply recurrent CDI per 1000 person-years increased by 42.7% (from 0.4408 to 0.6289 case) and 188.8% (from 0.0107 to 0.0309 case), respectively
Olsen et al,^30^ 2016	5 Databases: Medicare 5% Sample, Healthcare Cost and Utilization Project State Inpatient Databases and the National Inpatient Sample, OptumInsight Retrospective Database, and Premier Perspective	2000-2012	Not stated	*ICD-9*	Adults aged <65 y, 66.0 cases/100 000 person-years for OptumInsight Retrospective Database and 37.5 cases/100 000 person-years for State Inpatient Databases; adults aged >65 y, 677 cases/100 000 person-years for Medicare and 383 cases/100 000 person-years for State Inpatient Databases
Rabatsky-Ehr et al,^31^ 2008	Connecticut Department of Health reportable conditions surveillance system	2006	28 Hospitals and US Census for Connecticut	Clinical findings, laboratory tests	6.9 Cases/100 000 population
Kuntz et al,^32^ 2012	Kaiser Permanente Colorado and Kaiser Permanente Northwest (both inpatient and outpatient)	2007	Not stated	*ICD-9* code and positive test result needing antibiotic dispensation	14.9 Cases/10 000 patient-years; for women, 213 cases/100 000 enrollees aged 60-69 y, 420 cases/100 000 enrollees aged 70-79 y, and 795 cases/100 000 enrollees aged ≥80 y; for men, 167 cases/100 000 enrollees aged 60-69 y, 311 cases/100 000 enrollees aged 70-79 y, and 871 cases/100 000 enrollees aged ≥80 y
Lessa et al,^33^ 2014	Centers for Disease Control and Prevention Emerging Infections Program	2010	CDI surveillance in selected counties across 7 US states	Laboratory test (nucleic acid amplification)	Crude incidence varied by geographic area; community-associated, 30.7-41.3 cases/100 000 population; health care–associated, 58.5-94.8 cases/100 000 population
Reveles et al,^34^ 2017	Veterans Affairs Informatics and Computing Infrastructure	2002-2014	150 VHA hospitals and 820 VHA clinics	*ICD-9* and positive test result for CDI	Overall, 3.1 cases/10 000 VHA enrollees; 2002, 1.6 cases/10 000 VHA enrollees; 2013, 5.1 cases/10 000 VHA enrollees; 2014, 4.6 cases/10 000 VHA enrollees
Rhee et al,^35^ 2014	Centers for Disease Control and Prevention Emerging Infections Program	2010-2011	CDI surveillance in Monroe County, New York	Clinical diagnosis plus laboratory tests; enzyme immunoassay toxin or glutamate dehydrogenase with enzyme immunoassay toxin or nucleic acid amplification test	2010, 33.8 cases/100 000 population; 2011, 45.8 cases/100 000 population
Sanchez et al,^36^ 2005	Adult or adolescent spectrum of HIV disease project (inpatient and outpatient)	1992-2002	>100 Hospitals	Clinical findings, laboratory tests	All patients with HIV or AIDS, 4.12 cases/1000 person-years; patients with immunologic AIDS, 2.10 cases/1000 person-years; patients with clinical AIDS, 9.59 cases/1000 person-years
Troppy et al,^37^ 2019	3 Sources of data: Massachusetts Virtual Epidemiology Network, National Healthcare Safety Network, and 2010 US Census data in Massachusetts	2016	Not stated	Laboratory tests	132.5 Cases/100 000 population
Wendt et al,^38^ 2014	Centers for Disease Control and Prevention Emerging Infections Program in selected counties in 10 US states (California, Colorado, Connecticut, Georgia, Minnesota, New York, Oregon, Tennessee, Maryland, and New Mexico)	2010-2011	Not stated	Infection preventionist evaluated surveillance or diagnosis	Of 944 pediatric CDI cases identified, 71% were in California; CDI incidence children was highest among children aged 1 y (66.3 cases/per 100 000)
Young-Xu et al,^39^ 2015	VHA health care records	2009-2013	152 Hospitals	*ICD-9* and positive test for CDI	Overall CDI rate increased by 8.4% from 193 episodes/100 000 patient-years in 2009 to 209 episodes/100 000 patient-years in 2013
Denominator: geographic population (only inpatient)					
Argamany et al,^40^ 2015	US National Hospital Discharge Survey	2001-2010	National Hospital Discharge Survey data are collected manually or automatically by trained hospital staff, US Census Bureau staff, or National Center for Health Statistics staff	*ICD-9*	Pediatric population: 1.2 CDI discharges/1000 total discharges
Zilberberg et al,^41^ 2008	AHRQ National Inpatient Sample infant patients	2000-2005	Not stated	*ICD-9*	2000, 2.8 Cases/10 000 hospitalizations in infants; 2005, 5.1 cases/10 000 hospitalizations in infants
Zilberberg et al,^42^ 2008	AHRQ National Inpatient Sample adult patients	2000-2005	Not stated	*ICD-9*	2000, 5.5 Cases/10 000 hospitalizations in adults; 2005, 11.2 cases/10 000 hospitalizations in adults

Abbreviations: AHRQ, Agency for Healthcare Research and Quality; CDI, *Clostridium difficile* infection; *ICD-9*, *International Classification of Diseases, Ninth Revision*; VHA, Veterans Health Administration.

Three studies^40,41,42^ included only inpatients (Table 2). Two of these studies^41,42^ assessed the Agency for Healthcare Research and Quality (AHRQ) National Inpatient Sample (NIS). One evaluated infant patients from the AHRQ NIS cohort,^41^ and the other study evaluated adult patients from the AHRQ NIS cohort.^42^ Both studies documented substantial increases in CDI incidence between 2000 and 2005, from 2.8 to 5.1 cases per 10 000 hospitalizations, and from 5.5 to 11.2 cases per 10 000 hospitalizations, respectively.^41,42^ The third study,^40^ which was from the US National Hospital Discharge Survey between 2001 and 2010, found that the incidence of CDI in the pediatric population was 1.2 CDI discharges per 1000 total discharges.

### Incident Cases of CDI (36 Studies)

Twenty-six studies^43,44,45,46,47,48,49,50,51,52,53,54,55,56,57,58,59,60,61,62,63,64,65,66,67,68^ documented HO-CDIs, which we assumed were incident cases (Table 3). Of these studies, the AHRQ NIS was the main data set, represented by 10 included studies.^43,45,47,50,51,56,58,59,61,68^ These studies assessed diverse patient populations with different comorbidities, including peripartum women^68^ and patients with inflammatory bowel disease,^43^ lymphoma,^45^ leukemia,^58^ subarachnoid hemorrhage treated with microsurgical or endovascular aneurysm repair,^47^ chronic liver disease,^50^ hematopoietic stem cell transplant,^51^ megacolon,^56^ or heart failure.^59^ Thus, the results of these studies were also too diverse to pool together. One study^68^ found that the CDI incidence among peripartum women increased from 0.36 cases per 10 000 in 1998 to 0.70 cases per 10 000 in 2006. The US National Hospital Discharge Survey database was represented in 6 included studies.^49,52,53,55,64,65^ These studies also assessed diverse patient populations, including children^52^ and adults with different comorbidities, such as cancer^49,52^ and inflammatory bowel disease.^65^ In 1 of these studies,^65^ the overall incidence of HO-CDI was 369.8 cases per 10 000 hospitalizations for inflammatory bowel disease. In that same study,^65^ the HO-CDI incidence was 445.6 cases per 10 000 hospitalizations for ulcerative colitis and 220.3 cases per 10 000 hospitalizations for Crohn disease.

**Table 3.** Multicenter Studies (≥5 Sites) That Evaluated *Clostridium difficile* Infection Incidence Using Incident Cases

Source	Data Set	Study Period	Facilities or Hospitals, No.	*C difficil*e Definition	Incidence
HO infections					
Barber et al,^43^ 2018	AHRQ NIS patients with inflammatory bowel disease	1998-2014	Approximately 1000 hospitals	*ICD-9*	Incidence of HO-CDI, 7.8 cases/1000 hospitalizations in 1998 and 32.1 cases/ 1000 hospitalizations in 2014 among patients with Crohn disease, and 23.0 cases/1000 hospitalizations in 1998 and 84.7 cases/1000 hospitalizations in 2014 among patients with ulcerative colitis
Barlam et al,^44^ 2018	Truven Health Marketscan Commercial Claims and Encounters database	2011-2013	This database represents approximately 50 million covered lives (annually) for employed subscribers aged <65 y and their dependents	*ICD-9*	4 080 597 Unique individuals aged 1-64 y were admitted to the hospital in 2011; 12 025 had ≥1 *C difficile* diagnosis and complete enrollment information for 2011 (12 025 / 4 080 597 = 0.29%)
Bhandari et al,^45^ 2018	AHRQ NIS database	2007-2011	20% Stratified sample of US community hospitals	*ICD-9*	Incidence of HO-CDI was 2.13% among patients with lymphoma and 0.8% among patients without lymphoma
Brown et al,^46^ 2017	VA health care system	January 2006- December 2012	131 Acute care facilities	Laboratory tests	15.6 CDI cases/10 000 person-days
Dasenbrock et al,^47^ 2016	AHRQ NIS patients with subarachnoid hemorrhage who underwent microsurgical or endovascular aneurysm repair	2002-2011	Approximately 1000 hospitals	*ICD-9*	Incidence of HO-CDI was 1.9%
Davis et al,^48^ 2018	Electronic medical record of the health system	2014-2016	5-Hospital health system in Houston, Texas	Laboratory tests	Incidence of HO-CDI was 1.52%
Delgado et al,^49^ 2017	US NHDS	2001-2010	Not stated	*ICD-9*	Incidence of HO-CDI was 8.6 cases/1000 cancer discharges
Dotson et al,^50^ 2018	AHRQ NIS patients with chronic liver disease	2009	Approximately 1000 hospitals	*ICD-9*	Incidence of HO-CDI was 189.4 cases/10 000 discharges
Guddati et al,^51^ 2014	AHRQ NIS database	2000-2009	20% Stratified sample of US community hospitals	*ICD-9*	Incidence of HO-CDI among hematopoietic stem cell transplant recipients was 4.7%; nontransplant discharges were 0.86 cases/100 hospitalized patients
Gupta et al,^52^ 2016	US NHDS	2005-2009	Not stated	*ICD-9*	Overall HO-CDI incidence in children was 33.5 cases/10 000 hospitalizations
Gupta et al,^53^ 2017	US NHDS	2001-2010	100 Hospitals	*ICD-9*	Incidence of HO-CDI in patients with cancer was 64.7 cases/10 000 discharges in 2001-2002 and 109.1 cases/10 000 discharges in 2009-2010
Jiang et al,^54^ 2013	Rhode Island Hospital Discharge Database	2010-2011	11 Hospitals	*ICD-9* excluding present on admission code	HO-CDI, 1211 infections among 225 999 discharges = 53.5 cases/10 000 discharges
Khanna et al,^55^ 2016	US NHDS	2005-2009	100 Hospitals	*ICD-9*	HO-CDI incidence was 77.8 cases/10 000 hospitalizations
Kuy et al,^56^ 2016	AHRQ NIS patients with both *C difficile* and megacolon	2000-2010	Approximately 1000 hospitals	*ICD-9*	Overall incidence of megacolon among all hospitalized patients was 0.02% from 2000 to 2010; percentage of cases of megacolon due to CDI was 3.61% in 2000 and 9.39% in 2010
Lessa et al,^57^ 2015	Centers for Disease Control and Prevention Emerging Infections Program	2011	10 Program sites across 34 counties	Laboratory tests	453 000 Incident infections
Luo et al,^58^ 2015	AHRQ NIS patients with CDI with leukemia	2005-2011	Approximately 1000 hospitals	*ICD-9*	Overall incidence of CDI among patients with leukemia, 3.4%; incidence of CDI among all hospitalized patients, 0.85%; incidence of CDI among patients with leukemia in 2005, 3.0%; incidence of CDI among patients with leukemia in 2011, 3.5%
Mamic et al,^59^ 2016	AHRQ NIS database	2012	20% Stratified sample of US community hospitals	*ICD-9*	HO-CDI incidence among patients with a discharge diagnosis of heart failure, 3.5%
Miller et al,^60^ 2016	Healthcare Cost and Utilization Project State Inpatient Database for California	2005-2011	480 Hospitals	*ICD-9*	Overall incidence of HO-CDI, 0.15 cases/100 patients
Miller et al,^61^ 2016	AHRQ NIS database	2009-2011	480 Hospitals	*ICD-9*	HO-CDI incidence, 0.85 cases/100 patients in 2009, 0.89 cases/100 patients in 2010, and 0.99 cases/100 patients in 2011
Pant et al,^62^ 2016	Kids’ Inpatient Database (Healthcare Cost and Utilization Project)	2003-2012	Contains data from a variety of hospitals, including nonfederal, short-term, general, and special hospitals (including children’s hospitals) accessible by the general public	*ICD-9*	Incidence rate of CDI increased from 24.0 to 58.0 cases/10 000 discharges per year (*P* < .001) across all age groups, with the greatest increase in children aged ≥15 y
Pant et al,^63^ 2016		2012			Rate of CDI infection in children without solid-organ transplant was 0.6% and was greater (3.6%) in children with solid-organ transplant
Reveles et al,^64^ 2014	US NHDS of hospitalized adults	2001-2010	100 Hospitals	*ICD-9*	Incidence of HO-CDI, 4.5 cases/1000 adult discharges in 2001 and 8.2 cases/1000 adult discharges in 2010
Saffouri et al,^65^ 2017	US NHDS inflammatory bowel disease hospitalizations	2005-2009	100 Hospitals	*ICD-9*	Overall incidence of HO-CDI was 369.8 cases/10 000 inflammatory bowel disease hospitalizations; HO-CDI incidence was 445.6 cases/10 000 ulcerative colitis hospitalizations and 220.3 cases/10 000 Crohn disease hospitalizations
Sammons et al,^66^ 2013	Pediatric Health Information System Database	2006-2011	41 Pediatric hospitals	*ICD-9* and positive test for CDI	5107 Cases/693 516 patients; 73.6 cases/10 000 patients
Murphy et al,^67^ 2012	California hospital discharge data	2000-2007	29 Hospitals	*ICD-9*	28.7 Cases/10 000 admissions in 2000 and 52.2 cases/10 000 admissions in 2007
Kuntz et al,^68^ 2010	AHRQ NIS women hospitalized for childbirth and delivery	1998-2006	20% Stratified sample of discharges from nonfederal acute care hospitals	*ICD-9*	CDI incidence ranged from 0.36 CDI cases/10 000 peripartum women in 1998 to 0.70 CDI cases/10 000 peripartum women in 2006
Denominator: surgical patients					
Aquina et al,^69^ 2016	Statewide Planning and Research Cooperative System (a hospital discharge database by the New York Department of Health)	2005-2013	Patient-level data for all hospital admissions, ambulatory surgery procedures, and emergency department visits within New York State	*ICD-9*	22 Cases of CDI/1000 discharges
Bovonratwet et al,^70^ 2018	American College of Surgeons National Surgical Quality Improvement Program database	2015	500 Institutions	Clinical findings, laboratory tests	0.11% of the population had postoperative CDI
Bovonratwet et al,^71^ 2018	American College of Surgeons National Surgical Quality Improvement Program database	2015	500 Institutions	Clinical findings, laboratory tests	A total of 73 patients had *C difficile* colitis, generating an incidence of 1.05% (adult elderly, surgical patients [hip fracture])
Bovonratwet et al,^72^ 2018					The incidence of *C difficile* colitis was 0.10% (adult nonelderly and elderly, surgical patients [hip and knee arthroplasty])
Delanois et al,^73^ 2018	AHRQ NIS database	2009-2013	Not stated	*ICD-9*	After revision total hip arthroplasty, 1.7% of patients had postoperative CDI
Englesbe et al,^74^ 2010	Michigan Surgical Quality Collaborative and American College of Surgeons-National Surgical Quality Improvement Program on colectomy operations	2007-2009	24 Hospitals	Not stated	Among patients undergoing colectomies who received nonabsorbable antibiotics for bowel preparation, 1.9% had postoperative CDI; among patients undergoing colectomies who did not receive nonabsorbable antibiotics for bowel preparation, 3% had postoperative CDI
Lesperance et al,^75^ 2011	AHRQ NIS patients who underwent elective colon resections	2004-2006	Approximately 1000 hospitals	*ICD-9*	Overall, 1.4%; 2004, 1.31%; 2005, 1.45%; 2006, 1.67%
Guzman et al,^76^ 2016	AHRQ NIS patients who underwent cervical spine surgery	2002-2011	Approximately 1000 hospitals	*ICD-9*	Overall incidence of CDI in postoperative cervical spine surgery hospitalizations, 0.08%; in 2011, 0.14%
Gwam et al,^77^ 2018	AHRQ NIS database	2009-2013	Not stated	*ICD-9*	Incidence of CDI after revision total knee arthroplasty, 1.0%
Maltenfort et al,^78^ 2013	AHRQ NIS database	2002-2010	Not stated	*ICD-9*	Incidence of *C difficile* remained <0.6% during the study period

Abbreviations: AHRQ, Agency for Healthcare Research and Quality; CDI, *Clostridium difficile* infection; HO, hospital onset; *ICD-9*, *International Classification of Diseases, Ninth Revision*; NHDS, National Hospital Discharge Survey; NIS, National Inpatient Sample; VA, Veterans Affairs.

Ten studies^69,70,71,72,73,74,75,76,77,78^ evaluated surgical patients (Table 3), and, thus, we assumed that the CDI cases were incident cases. Five studies^73,75,76,77,78^ used data from AHRQ NIS. These AHRQ NIS studies analyzed a variety of surgical procedures, including spine surgery^76^; hip,^73^ knee,^77^ or lower-extremity^78^ arthroplasty; and elective colon resections.^75^ One of them had CDI occurring in 1.4% of patients, for a rate of 144.99 cases of *C difficile* colitis per 10 000 elective colon resections, and the incidence increased from 1.31% in 2004 to 1.67% in 2006.^75^

### LOS Associated With CDI (20 Studies)

Twenty studies^16,54,66,79,80,81,82,83,84,85,86,87,88,89,90,91,92,93,94^ (Table 4) evaluated CDI-associated LOS. Sixteen studies^54,66,79,80,81,82,83,84,85,86,87,88,89,92,94,95^ used propensity score matching to evaluate LOS associated with CDI, 2 studies^16,93^ used postinfection LOS, 1 study^90^ matched on LOS from admission until either positive *C difficile* test results or discharge, and 1 study^91^ accounted for time to infection using a multistate model. Also, one of the propensity score matched–studies applied multistate modeling to account for timing of infection.^88^ Pediatric patients were included in 3 of these studies.^66,86,87^

**Table 4.** Length of Stay Associated With *Clostridium difficile* Infection Among Studies That Used Appropriate Methods^a^

Source	Data Set	Study Period	Patient Population	Facilities or Hospitals, No.	LOS	Method	Downs and Black Score^b^
Campbell et al,^79^ 2013	Cerner Health Facts Electronic Health Record Database	2005-2011	Hospitalized adults at high risk for poor outcomes including those aged >65 y, those with complex conditions or chronic diseases (renal disease, cancer, inflammatory bowel disease) and those with concomitant antibiotic use	74	Among patients aged >65 y with HO-CDI, mean 19.10 d; among patients without CDI aged >65 y, mean, 16.06 d; mean difference, 3.04 d (95% CI, 1.44-4.63 d)	Propensity score matched including matching on preinfection LOS	17
Drozd et al,^80^ 2015	Medicare Standard Analytic Files	2009-2010	Inpatients	5% Random sample of Medicare	Among patients with CDI, mean, 7.0 d; among patients without CDI, mean, 3.8 d; mean difference, 3.2 d	Propensity score matched	17
Dubberke et al,^81^ 2008	Barnes-Jewish Hospital	2003	Inpatients	1	Among patients with CDI, median, 9.6 d; among patients without CDI, median, 5.8 d; attributable median difference, 2.8 d	Propensity score matched	15
Dubberke et al,^16^ 2010	Hospitals in Centers for Disease Control and Prevention Epicenter Network	July 2000 to June 2006	Hospitalized adults	5 Hospitals	Community-onset, patients with community-associated CDI, median, 5 d; patients with community-onset HCF-associated CDI (study hospital), median, 6 d; patients with community-onset HCF-associated CDI (other hospital), median, 8 d	Postinfection LOS	13
Egorova et al,^82^ 2015	AHRQ NIS database	2000-2011	Patients included in the Nationwide Inpatient Sample	20% of US Hospitals	Among patients with CDI, median (IQR), 15 (9-25) d; among patients without CDI, median (IQR), 8.3 (4.6-13.6) d; attributable median difference, 6.7 d	Propensity score matched	17
Gabriel et al,^83^ 2018	University of California Irvine Trauma Database	2014-2016	CDI in hospitalized adult trauma patients	1	Odds ratio, 1.39; 95% CI, 1.16-1.66	Propensity score matched	15
Jiang et al,^54^ 2013	Rhode Island Hospital Discharge Database	2010-2011	Hospitalized adults; evaluated health care–onset CDI	11	Among patients with CDI, mean (SD), 18.9 (21.7) d; among patients without CDI, mean (SD), 8.6 (11.3) d; mean difference, 10.3 d	Propensity score matched	15
Li et al,^84^ 2016	Veterans Affairs Surgical Quality Improvement Program database and Decision Support System pharmacy	2009-2013	Postoperative adult patients	134	Among patients with CDI, mean (SD), 15.6 (19.5) d; among patients without CDI, mean (SD), 8.1 (12.6) d; mean difference, 7.5 d	Propensity score matched	18
Magee et al,^85^ 2015	Discharges from Premier database	2009-2011	Inpatients	Geographically diverse hospitals	Among patients with CDI mean (SD), 14.4 (18.3) d; among patients without CDI, mean (SD), 8.7 (15.6) d; mean difference, 5.7 d	Propensity score matched	17
Mehrotra et al,^86^ 2017	AHRQ Kids’ Inpatient Database	2012	Pediatric inpatients	2500-4100 Hospitals/y	Among patients with CDI mean, 9.4 d (95% CI, 9.1-9.6 d); among patients without CDI, mean, 5.4 d (95% CI, 5.3-5.6 d); mean difference, 3.9 d	Propensity score matched	17
Nylund et al,^87^ 2011	Healthcare Cost and Utilization Project Kids’ Inpatient Database	1997, 2000, 2003, 2006	Pediatric patients	Not stated	Odds ratio, 4.34; 95% CI, 3.97-4.83	Propensity score matched	19
Pak et al,^88^ 2017	Mount Sinai Hospital Electronic Medical Record	2009-2015	Adult inpatients	1	Median difference by case definition: *ICD-9* code, 3.1 d (95% CI, 2.2-3.9 d); positive toxin enzyme immunoassay, 10.1 d (95% CI, 7.3-12.2 d); positive toxin polymerase chain reaction, 6.6 d (95% CI, 5.0-8.1 d); either toxin assay, 7.2 d (95% CI, 5.8-8.3 d); by any of these, 5.7 d (95% CI, 4.5-6.6 d); stratification by time to first positive toxin assay, 3.1 d (95% CI, 1.7-4.4 d); under the same case definition, the multistate model averaged an excess LOS of 3.3 d (95% CI, 2.6-4.0 d)	Propensity score matched plus multistate modeling to account for timing of infection	14
Radcliff et al,^89^ 2016	Texas Health Care Information Collection Inpatient Public Use Data Files	2007-2011	Inpatients	Texas hospitals	For 2007, among patients with CDI, mean, 19.0 d; among patients without CDI, mean, 9.7 d; mean difference: 9.3 d; for 2011, among patients with CDI, mean, 16.5 d; among patients without CDI, mean, 9.2 d; mean difference, 7.4 d	Propensity score matched	12
Sammons et al,^66^ 2013	Pediatric Health Information System Database	2006-2011	Hospitalized children at 41 children’s hospitals	41	Among patients with HO-CDI, median (IQR), 23 d (12-44 d); among patients without CDI matched to patients with HO-CDI, median (IQR not stated), 4 d; median difference, 19 d; adjusted mean difference, 21.6 d (95% CI, 19.29-23.90 d)	Propensity score matched	15
Song et al,^90^ 2008	Johns Hopkins Hospital	January 2000 to October 2005	Hospitalized adults patients	1	Among patients with CDI, median, 19 d; among patients without CDI, median, 18 d; adjusted difference, 13% increased LOS among patients with CDI	Matched on LOS from admission to either positive *C difficile* test or discharge	15
Stevens et al,^91^ 2015	VA Healthcare System	January 2005 to December 2012	Hospitalized adults patients	120 Acute care facilities	Among patients with CDI, mean (SD), 19.4 (31.7) d; among patients without CDI, mean (SD), 5.4 (8.4) d; mean difference, 14 d; multistate modeling estimated an attributable LOS of only 2.27 d (95% CI, 2.14-2.40 d)	Multistate modeling to account for timing of infection	19
Stewart et al,^92^ 2011	AHRQ NIS database	2007	Patients included in the Nationwide Inpatient Sample; age unknown, assumed all ages	20% of US hospitals	Among patients with CDI, mean (SD), 13.0 (14) d; among patients without CDI mean (SD), 7.9 (9) d; mean difference, 5.1 d	Propensity score matched	17
Stewart et al,^93^ 2012	Pennsylvania State College of Medicine	2004-2009	Patients with and without hematologic malignancies who acquired CDI	1	Postinfection LOS for patients with CDI with malignancies and receiving chemotherapy, mean (SD), 22.4 (23.2) d; postinfection LOS for patients with CDI without malignancies, mean (SD), 10.2 (10) d	Postinfection LOS	14
Tabak et al,^94^ 2013	CareFusion database of 6 Pennsylvania hospitals	2007-2008	Hospitalized patients	6	Among patients with CDI, mean (SD), 16.3 (14.2) d; among patients without CDI, mean (SD), 14.0 (11.9) d; attributable days, 2.4 (95% CI, 0.7-4.4; *P* < .01)	Propensity score matched	18
Zilberberg et al,^95^ 2009	AHRQ NIS database	2005	Hospitalized patients	Approximately 1000 hospitals	Patients with CDI had an independent increase in the hospital LOS by 6.1 d (95% CI, 4.9-7.4 d)	Propensity score matched	16

Abbreviations: AHRQ, Agency for Healthcare Research and Quality; CDI, *Clostridium difficile* infection; HCF, health care facility; HO, hospital onset; *ICD-9*, *International Classification of Diseases, Ninth Revision*; IQR, interquartile range; LOS, length of stay; NIS, National Inpatient Sample; VA, Veterans’ Affairs.

^a^ Methods include propensity score matching or postinfection LOS or matched on preinfection LOS or multistate modeling.

^b^ The Downs and Black scale measures study quality, with a score of 18 or higher indicating higher quality, and a maximum score of 28 possible.^10^

Among the 13 propensity score–matched studies of adults, the CDI-associated mean difference in LOS (in days) between patients with CDI and patients who did not have CDI varied greatly from 3.0 days (95% CI, 1.44-4.63 days)^79^ to 10.3 days.^54^ Among the 3 pediatric propensity score–matched studies,^66,86,87^ the highest CDI-associated mean difference in LOS (in days) was 21.6 days (95% CI, 19.29-23.90 days).^66^

Among the studies that used multistate models to account for timing of infection, a study^91^ performed in the Veterans Affairs health care system found that the magnitude of its estimated impact was smaller when methods were used to account for the time-varying nature of infection. That study estimated a CDI-attributable LOS of only 2.27 days (95% CI, 2.14-2.40 days).^91^ The other study^88^ that performed propensity score matching and used a multistate model converged on similar excess LOS estimates of 3.1 days (95% CI, 1.7-4.4 days) and 3.3 days (95% CI, 2.6-4.0 days), respectively.

Four studies^84,87,91,94^ that evaluated LOS earned 18 or more points on the Downs and Black scale.^10^ One study^91^ also used multistate modeling. Another was also performed in the Veterans Affairs health care system^84,91^ and found a mean difference between patients with and without CDI of 7.5 days.^84^ One study^87^ of pediatric patients found that those with CDI had a longer LOS (adjusted odds ratio, 4.34; 95% CI, 3.97-4.83). Another study^94^ of adult patients in Pennsylvania hospitals showed an attributable hospital LOS difference of 2.4 days (95% CI, 0.7-4.4 days; *P* < .01) between patients with and without CDI.

## Discussion

National epidemiological investigations have demonstrated recent marked increases in CDI in the United States.^34^ Thus, a national public health response to this increase requires current estimates of the CDI incidence.^96,97,98^ Our systematic review of the literature found that the CDI incidence varied by study and that the investigators used different denominators when they calculated the incidence for specific study populations. In our meta-analysis of studies that used patient-days as the denominator, we estimated the incidence of CDI in the United States to be 8.3 CDI cases per 10 000 patient-days.

Variation in CDI incidence may be due, in part, to advances in diagnostic technology and variations in diagnostic practices.^99,100,101^ Nucleic acid amplification tests are more sensitive than traditional *C difficile* stool tests (eg, toxin enzyme immunoassay). Nucleic acid amplification tests have been used more frequently in clinical practice since 2009, when the first commercial polymerase chain reaction was approved by the US Food and Drug Administration.^102^ The topic of CDI testing methods and risk adjustment is complex.^103,104^ Concerns have been expressed about the adequacy of risk adjustment to account for different CDI testing methods (toxin enzyme immunoassay alone, polymerase chain reaction alone, toxin enzyme immunoassay plus glutamate dehydrogenase followed by polymerase chain reaction for discrepancies, polymerase chain reaction followed by toxin enzyme immunoassay, and other diagnostic options) across HCFs. The choice of testing methods substantially affects the performance of these testing algorithms.^99,100,101^

In addition, the CDI incidence found by these studies likely varied because of the different database structures adopted by the various hospitals.^13,14,15,16,17,18,19,20,21,22,23,24,25,26,27,28,29,30,31,32,33,34,35,36,37,38,39,40,41,42,43,44,45,46,47,48,49,50,51,52,53,54,55,56,57,58,59,60,61,62,63,64,65,66,67,68,69,70,71,72,73,74,75,76,77,78^ Some analyses were based on health care systems databases, but most used large infection control surveillance, state, or national discharge databases.^13,14,15,16,17,18,19,20,21,22,23,24,25^ Beginning in January 2013, the Centers for Medicare & Medicaid Services began requiring public reporting of CDI rates via the National Healthcare Safety Network for those hospitals participating in the Inpatient Prospective Payment System.^64^ Specifically, 1 study^29^ demonstrated an increase in the annual incidence of CDI and multiply recurrent CDI per 1000 person-years by 42.7% and 188.8%, respectively, between 2001 and 2012. Another CDI surveillance study^33^ in 7 US states reported an increase not only in community-associated CDI incidence rates but also an increase in health care–associated CDI incidence rates. Furthermore, CDI can complicate comorbid conditions and result in the need for additional hospital resources.^34^ Included studies detected an increase in the CDI incidence in patients with inflammatory bowel disease,^43^ patients with cancer,^52^ those undergoing surgery,^75,76^ and even infants.^41^ The results of our systematic review of literature and meta-analysis emphasize the need to perform *C difficile* surveillance and direct resources to the prevention of CDI in order to reduce the incidence across the United States.

### Limitations

This systematic literature review has some limitations. First, the results of systematic literature reviews and meta-analyses are only as valid as the results of the studies evaluated. Most studies included in this systematic literature review were of moderate-to-low quality and may have overestimated the outcomes. We need more high-quality studies so that we can accurately determine postinfection LOS, because LOS before the infection should not be attributed to *C difficile*.^5^ Second, we included studies that used *ICD-9* codes to define CDI. The *ICD-9* codes are used for billing purposes and are not ideal for surveillance. However, a prior meta-analysis^105^ found that the *ICD-9* code for *C difficile* had good sensitivity, specificity, positive predictive value, and negative predictive value compared with clinical definitions. Third, we only included studies conducted in the United States and published in English, which limits the external validity of this research. We used these inclusion criteria because our goal was to evaluate the burden of CDI in the United States. Future systematic literature reviews should be performed to evaluate this burden in other countries. Fourth, we found heterogeneity in all LOS-stratified analyses (eAppendix 2 and eTable in the Supplement). We found that the higher-quality studies that used advanced statistical methods to attempt to account for time-dependent bias found lower CDI-attributable LOS compared with other studies that did not use advanced methods. In addition, our incidence estimates were derived from multicenter studies only. Incidence rates in small studies may be variable and subject to bias; thus, this criterion was established a priori to determine representative incidence rates. From incident cases of CDI (36 studies), we were unable to exclude recurrent and multiply recurrent CDI cases if the study did not exclude those cases. For this meta-analysis, we decided to calculate the incidence rate with studies with a similar denominator (patient-days), with a result of 8.3 CDI cases per 10 000 patient-days.

## Conclusions

Pooled estimates from the currently available literature suggest that *C difficile* is associated with a large burden on the US health care system. However, these estimates should be used with caution, and higher-quality studies should be completed to guide future evaluations of *C difficile* prevention and treatment interventions.

## Notes

Supplement. **eAppendix 1. **Search Methods **eAppendix 2.** Statistical Methods **eReferences.** **eTable. **Subset Analyses Evaluating Hospital Length of Stay Attributable to *Clostridium difficile *Infection (8 Studies)
