# Supplementary material for: Incidence and Outcomes Associated With Clostridium difficile Infections: A Systematic Review and Meta-analysis
Source: JAMA Netw Open. 2020 Jan 8;3(1):e1917597. doi: 10.1001/jamanetworkopen.2019.17597 (PMC6991241; doi:10.1001/jamanetworkopen.2019.17597)
Supplement: Supplement. — eAppendix 1. Search Methods eAppendix 2. Statistical Methods eReferences. eTable. Subset Analyses Evaluating Hospital Length of Stay Attributable to Clostridium difficile Infection (8 Studies) [file jamanetwopen-3-e1917597-s001.pdf]

Supplementary Online Content

Marra AR, Perencevich EN, Nelson RE, et al. Incidence and outcomes associated with *Clostridium difficile* infections: a systematic review and meta-analysis. *JAMA Netw Open*. 2020;3(1):e1917597. doi:10.1001/jamanetworkopen.2019.17597

eAppendix 1. Search Methods

eAppendix 2. Statistical Methods

eReferences.

eTable. Subset Analyses Evaluating Hospital Length of Stay Attributable to *Clostridium difficile* Infection (8 Studies)

This supplementary material has been provided by the authors to give readers additional information about their work.

© 2020 Marra AR et al. *JAMA Network Open.*

eAppendix 1. Search Methods

Methods: An experienced health sciences librarian conducted systematic searches in MEDLINE via Ovid, Cochrane Library Databases via Wiley, CINAHL Complete via EBSCO, Scopus, and Web of Science to identify papers published from the inception of the database to February 2019. After the citations were entered into EndNote, citations older than 2000 were excluded. A combination of keywords and subject headings were used for Clostridium difficile, length of stay, and incidence. The full search strategies can be found in the appendix.

Ovid MEDLINE(R) In-Process & Other Non-Indexed Citations and Ovid MEDLINE(R) 1946 to Present (Run on February 21, 2019, 10957 Results)

1. exp Clostridium difficile/
2. exp Clostridium Infections/
3. (Clostridium difficile or Clostridium infection* or C difficile or Pseudomembranous Enterocolitis or Colitis Pseudomembranous or Pseudomembranous Colitis or Antibiotic-Associated Colitis or Clostridium Enterocolitis or Pseudomembranous Enteritis or Antibiotic Associated diarrhea or Antibiotic Associated diarrhea).tw.
4. 1 or 2 or 3
5. exp "Length of Stay"/
6. (length of stay* or length of hospital stay*).ti,ab.
7. ((hospital or hospitali?ed or bed) adj2 days).ti,ab.
8. 5 or 6 or 7
9. exp Epidemiology/
10. exp Epidemiologic Studies/
11. exp Incidence/
12. (incidence or occur* or frequenc* or proportion* or rate* or number* or percent*).ti,ab.
13. or/9-12
14. 8 or 13
15. 4 and 14

CINAHL Complete (Run on February 22, 2019, 3863 citations found)

1. MH "Clostridium Difficile" OR MH "Clostridium Infections+") OR (“Clostridium difficile” OR “Clostridium infection*” OR “C difficile” OR “C diff” OR CDI OR “Pseudomembranous

© 2020 Marra AR et al. *JAMA Network Open.*

Enterocolitis” OR “Colitis Pseudomembranous” OR “Pseudomembranous Colitis” OR “Antibiotic-Associated Colitis” OR “Clostridium Enterocolitis” OR “Pseudomembranous Enteritis” OR “Antibiotic Associated diarrhea” OR “Antibiotic Associated diarrhea”
2. TI ((length of stay* OR length of hospital stay*)) OR AB ((length of stay* OR length of hospital stay*))
3. (MH "Length of Stay")
4. TI (((hospital or hospitali#ed or bed) N2 days)) OR AB (((hospital or hospitali#ed or bed) N2 days))
5. S2 OR S3 OR S4
6. (MH "Epidemiology+")
7. (MH "Epidemiological Research+") OR (MH "Incidence")
8. AB ((incidence OR occur* OR frequenc* OR proportion* OR rate* OR number* OR percent*)) OR TI ((incidence OR occur* OR frequenc* OR proportion* OR rate* OR number* OR percent*))
9. S6 OR S7 OR S8
10. S5 OR S9
11. S1 AND S10

Cochrane Databases via Wiley (Run on February 25, 2019, 35 CDSR, 68 CENTRAL)

1. MeSH descriptor: [Clostridium difficile] explode all trees
2. MeSH descriptor: [Clostridium Infections] explode all trees
3. “Clostridium difficile” OR “Clostridium infection*” OR “C difficile” OR “Pseudomembranous Enterocolitis” OR “Colitis Pseudomembranous” OR “Pseudomembranous Colitis” OR “Antibiotic-Associated Colitis” OR “Clostridium Enterocolitis” OR “Pseudomembranous Enteritis” OR “Antibiotic Associated diarrhea” OR “Antibiotic Associated diarrhea”
4. #1 OR #2 OR #3
5. ("length of stay*" OR "length of hospital stay*"):ti,ab,kw
6. MeSH descriptor: [Length of Stay] explode all trees
7. (hospital or hospitalized or hospitalised or bed) NEAR/2 days
8. #5 OR #6 OR #7
9. MeSH descriptor: [Epidemiology] explode all trees
10. MeSH descriptor: [Incidence] explode all trees
11. MeSH descriptor: [Epidemiologic Studies] explode all trees
12. (incidence or occur* or frequenc* or proportion* or rate* or number* or percent*):ti,ab,kw
13. #9 OR #10 OR #11 OR #12
14. #4 AND #8 AND #13

Scopus (Search run on February 25, 2019, 11063 results.)

1. “Clostridium difficile” OR “Clostridium infection*” OR “C difficile” OR “Pseudomembranous Enterocolitis” OR “Colitis Pseudomembranous” OR “Pseudomembranous Colitis” OR “Antibiotic-Associated Colitis” OR “Clostridium Enterocolitis”
2. “Pseudomembranous Enteritis” OR “Antibiotic Associated diarrhea” OR “Antibiotic Associated diarrhea”
3. #1 OR #2
4. "length of stay*" OR "length of hospital stay*" or incidence or occur* or frequenc* or proportion* or rate* or number* or percent*
5. (hospital or hospitalized or hospitalised or bed) W/2 days

© 2020 Marra AR et al. *JAMA Network Open.*

6. #4 OR #6
7. #3 AND #6

Web of Science Core Collection (Used Topic field for all. Searched on February 27, 2019, 8789 Results)

1. “Clostridium difficile” OR “Clostridium infection*” OR “C difficile” OR “Pseudomembranous Enterocolitis” OR “Colitis Pseudomembranous” OR “Pseudomembranous Colitis” OR “Antibiotic-Associated Colitis” OR “Clostridium Enterocolitis” OR “Pseudomembranous Enteritis” OR “Antibiotic Associated diarrhea” OR “Antibiotic Associated diarrhea”
2. "length of stay*" OR "length of hospital stay*" or incidence or occur* or frequenc* or proportion* or rate* or number* or percent*
3. (hospital or hospitalized or hospitalised or bed) near/2 days
4. #2 OR #3
5. #1 AND #4

© 2020 Marra AR et al. *JAMA Network Open.*

eAppendix 2. Statistical Methods

In order to meta-analyze the extracted data, we calculated the mean LOS and standard deviation between CDI and non-CDI patients. We estimated the sample's mean and standard deviation from studies that reported median and inter-quartile range (IQR) LOS using the method devised by Hozo et al. [1]. We employed random-effect models to obtain pooled mean differences, using the Cochrane Review Manager (RevMan) version 5.3 [2]. To assess heterogeneity, we used the Cochran Q statistic, the I^2 statistic, and the results of stratified analyses based on the following *a priori* categories: age (*i.e.* pediatric, adult) and patient population (*i.e.* Agency for Healthcare Research and Quality [AHRQ] supported dataset, study quality, sample size of study).

On the basis of results from 8 studies, the pooled mean difference in LOS for patients with CDI and the controls was 5.58 days; 95% CI 4.76-6.39, which was statistically significant [54,80,84,82,85,86,92,94] (Figure 2). However, the result was heterogenous (heterogeneity $p<0.001$, $I^2=98\%$). We performed other stratified analyses considering other subgroups of patients with *C. difficile* infection and uninfected controls including AHRQ studies vs. non-AHRQ studies; higher quality studies (considering higher quality score≥18); adult patient studies vs. pediatric patient studies; cases; and sample size studies with >5,000 patients vs. <=5,000 patients for CDI cases (Table 3), but in all subgroups there was high heterogeneity. The funnel plot (appendix 2) of these studies was symmetrical with four studies on each side of the pooled mean difference and the Egger test for funnel plot asymmetry was not statistically significant (p=0.74), thus we did not find evidence of publication bias.

eReferences

1. Hozo SP, Djulbegovic B, Hozo I. Estimating the Mean and Variance from the Median, Range, and the Size of a Sample. *BMC Medical Research Methodology* 2005;5:13.
2. 2011. Review Manager (RevMan), Version 5.3 ed. The Nordic Cochrane Center, The Cochrane Collaboration, Copenhagen.

© 2020 Marra AR et al. *JAMA Network Open.*

eTable. Subset Analyses Evaluating Hospital Length of Stay Attributable to *Clostridium difficile* Infection (8 Studies)*

Subset	Number of Studies Included	Pooled Mean Difference in Hospital Length of Stay Comparing patients CDI with uninfected patients (95% confidence interval)	I^2 test for heterogeneity
AHRQ Supported Datasets	3	5.24 (4.14, 6.34) days	98%
Non-AHRQ Supported Datasets	5	5.82 (4.02, 7.64) days	98%
Higher quality studies	2	4.96 (-0.14, 10.05) days	95%
Lower Quality Studies	6	5.63 (4.75, 6.52) days	98%
Adult Patient Studies	7	5.32 (5.21, 5.42) days	98%
Pediatric Patient Studies	1	4.0 (3.71, 4.29) days	N/A
Studies with Sample Size >5,000 Patients	3	4.94 (4.14, 5.74) days	98%
Studies with Sample Size <5,000 Patients	5	6.03 (3.48, 8.57) days	98%

CDI - C. difficile infection; AHRQ – Agency for Healthcare Research and Quality

*Reasons for not including the other 12 LOS studies in the meta-analysis: Campbell 2013 [79]: no standard deviation (SD) in both groups (CDI vs. non-CDI); Dubberke 2008 (EID) [81]: interquartile range (IQR) not stated; Dubberke 2010 [16]: IQR not stated; Gabriel 2018 [83]:no mean and no SD in both groups (CDI vs. non-CDI); Nylund 2011 [87]: The sample is not stated for both groups (CDI vs. non-CDI); Pak 2015 [88]: no mean and SD for non–CDI group; Radcliff 2016 [89]: no SD for both groups (CDI vs. non-CDI); Sammons 2013 [66]: IQR not stated in non-CDI group; Song 2008 [90]: IQR not stated; Stewart 2012[93]: not stated non-CDI group; Zilberberg 2009 [95]: no mean and SD for non–CDI group; Stevens 2015 [91]: the difference of CDI and non-CDI patients (43,540 vs. 3,917,664) will not be accurate to pool these data in the meta-analysis. The authors [91] performed bootstrapping techniques from 1,000 runs of 3.96 million hospitalization each, to adjust it.

© 2020 Marra AR et al. *JAMA Network Open.*
